# Brain activity during dual-task standing in older adults

**DOI:** 10.1186/s12984-022-01095-3

**Published:** 2022-11-11

**Authors:** Melike Kahya, Natalia A. Gouskova, On-Yee Lo, Junhong Zhou, Davide Cappon, Emma Finnerty, Alvaro Pascual-Leone, Lewis A. Lipsitz, Jeffrey M. Hausdorff, Brad Manor

**Affiliations:** 1 Hinda and Arthur Marcus Institute for Aging Research, Hebrew SeniorLife, Boston, MA 02131 USA; 2 Beth Israel Deaconess Medical Center, Boston, MA USA; 3 Harvard Medical School, Boston, MA USA; 4 Deanna and Sidney Wolk Center for Memory Health, Hebrew SeniorLife, Boston, MA USA; 5 School of Medicine, Boston University, Boston, MA USA; 6 Guttman Brain Health Institute, Institut Guttmann de Neurorehabilitació, Barcelona, Spain; 7 Center for the Study of Movement, Cognition, and Mobility, Neurological Institute, Tel Aviv Sourasky Medical Center, Tel Aviv, Israel; 8 Sagol School of Neuroscience and Sackler Faculty of Medicine, Tel Aviv University, Tel Aviv, Israel; 9 Rush Alzheimer’s Disease Center and Department of Orthopedic Surgery, Rush University Medical Center, Chicago, IL USA

**Keywords:** Electroencephalography, Brain activity, Dual-tasking, Posture, Older adults

## Abstract

**Background:**

In older adults, the extent to which performing a cognitive task when standing diminishes postural control is predictive of future falls and cognitive decline. The neurophysiology of such “dual-tasking” and its effect on postural control (i.e., dual-task cost) in older adults are poorly understood. The purpose of this study was to use electroencephalography (EEG) to examine the effects of dual-tasking when standing on brain activity in older adults. We hypothesized that compared to single-task “quiet” standing, dual-task standing would decrease alpha power, which has been linked to decreased motor inhibition, as well as increase the ratio of theta to beta power, which has been linked to increased attentional control.

**Methods:**

Thirty older adults without overt disease completed four separate visits. Postural sway together with EEG (32-channels) were recorded during trials of standing with and without a concurrent verbalized serial subtraction dual-task. Postural control was measured by average sway area, velocity, and path length. EEG metrics included absolute alpha-, theta-, and beta-band powers as well as theta/beta power ratio, within six demarcated regions-of-interest: the left and right anterior, central, and posterior regions of the brain.

**Results:**

Most EEG metrics demonstrated moderate-to-high between-day test–retest reliability (intra-class correlation coefficients > 0.70). Compared with quiet standing, dual-tasking decreased alpha-band power particularly in the central regions bilaterally (p = 0.002) and increased theta/beta power ratio in the anterior regions bilaterally (p < 0.001). A greater increase in theta/beta ratio from quiet standing to dual-tasking in numerous demarcated brain regions correlated with greater dual-task cost (i.e., absolute increase, indicative of worse performance) to postural sway metrics (r = 0.45–0.56, p < 0.01). Lastly, participants who exhibited greater alpha power during dual-tasking in the anterior-right (r = 0.52, p < 0.01) and central-right (r = 0.48, p < 0.01) regions had greater postural sway velocity during dual-tasking.

**Conclusion:**

In healthy older adults, alpha power and theta/beta power ratio change with dual-task standing. The change in theta/beta power ratio in particular may be related to the ability to regulate standing postural control when simultaneously performing unrelated, attention-demanding cognitive tasks. Modulation of brain oscillatory activity might therefore be a novel target to minimize dual-task cost in older adults.

## Introduction

Many older adults exhibit deficits in postural control when dual-tasking, that is, standing while performing an unrelated cognitive task such as talking, reading, or making decisions [1–3]. Several longitudinal studies using laboratory-based assessments have demonstrated that older adults who exhibit greater dual-task cost (i.e., percent decrement) to measures of standing postural sway are not only more likely to fall [4], but also have an increased risk of significant cognitive decline and dementia in the future [5]. Postural control is regulated in part by the dynamic interplay between cortical and subcortical brain networks [6, 7]. Sensorimotor areas, and their distributed bi-hemispheric network, are engaged in pre-selecting and altering postural control strategies based upon the current environmental situation [8, 9]. The couplings between attentional capacity, working memory, and standing balance also suggest involvement of bilateral frontal cerebral cortices and prefrontal networks in the maintenance of postural control, particularly during simultaneous performance of unrelated cognitive tasks [10].

Despite the importance of dual-tasking in many daily life activities, the patterns of brain activation that enable older adults to maintain balance when dual-tasking are not completely understood. Recent research using EEG power spectrum analysis has demonstrated that in healthy young adults, standing within conditions that increasingly challenge postural control results in a progressive decrease in the absolute power in the alpha frequency band (8–16 Hz) within both sensorimotor and parietal cortical regions [11, 12]. Diminished alpha band power has also been linked to reductions in motor inhibition [13], which has in turn been associated with worse balance in older adults [14]. Recently, still other studies have linked the ratio of power in the theta band (4–7 Hz) to the power in the beta band (18–32 Hz) (i.e., the theta/beta power ratio) to attentional control [15] and performance in tasks requiring executive function [16, 17]. Together, this evidence suggests that the ability to maintain standing balance when dual-tasking is likely accompanied by changes in brain activity across multiple frequencies, and specifically in alpha power and theta/beta ratio. The EEG spectral power dynamics of dual-task standing, however, has yet to be studied in older adults.

The purpose of this study was to examine the effect of dual-tasking on EEG spectral power dynamics when standing in older adults without overt illness or disease. Based on prior literature, we focused on the absolute power in the alpha-, beta-, and theta-bands, as well as the theta/beta ratio, and further examined the test–retest reliability of these metrics. We hypothesized that in older adults, dual-tasking, as compared to quiet standing, would decrease alpha power and increase theta/beta ratio, since these two specific metrics have been previously linked to reduced motor inhibition and increased attentional control, respectively. We also hypothesized that the magnitude of dual task-induced change in these EEG metrics would correlate with the magnitude of the dual-task cost to postural sway outcomes.

## Methods

A secondary analysis was completed on data from a multi-site study testing the effects of single exposures to transcranial direct current stimulation targeting different brain regions on dual-task standing performance in older adults [18]. That study was approved by the Institutional Review Boards of Hebrew SeniorLife and Tel Aviv Sourasky Medical Center and conducted in accordance with the principles of the Declaration of Helsinki. All participants provided written informed consent at the beginning of their first study visit. For the current analysis, we focused on the pre-stimulation dual-task standing assessments that were completed during four laboratory visits each separated by approximately one week. The analysis was further limited to data from the participants tested at the Hebrew SeniorLife site because only this site included EEG recordings.

### Subjects

Thirty older adults were recruited from the community and tested at Hebrew SeniorLife’s Hinda and Arthur Marcus Institute for Aging Research (Boston, MA, U.S). Participants were included in the study if they were persons aged 65 and older, able to read, write and communicate in English, and able to stand and walk without use of an assistive device. Exclusion criteria included a Montreal Cognitive Assessment (MoCA) [19] score < 24, self-reported presence of neurodegenerative conditions such as Parkinson’s disease or multiple sclerosis, self-report of acute illness, injury or other unstable medical condition and hospitalization within the past three months; self-reported active cancer or other terminal diseases; any report of severe lower-extremity arthritis, pain or orthopedic problems that would likely affect gait or standing balance; physician-diagnosis of peripheral neuropathy, or other peripheral neuromuscular disease; use of antipsychotics, anti-seizure, or other neuroactive medications; any report or physician-diagnosis of schizophrenia or other psychiatric illness.

### Experimental protocol

All participants completed an initial screening and baseline visit. After providing informed consent, the research staff recorded the individual’s demographics, height, weight, medical history, and medications. The MoCA was then completed. Interested and eligible participants were enrolled in the study and completed the Timed Up and Go test of mobility. They were then scheduled to complete four additional in-person experimental visits during which EEG and postural sway were recorded during a dual-task standing assessment.

At the beginning of each experimental visit, participants were outfitted with wearable motion sensors (APDM, Portland, OR) [26] and a 32-channel EEG system (Starstim Enobio 32, Neuroelectrics Inc., Cambridge, MA)**.** The motion sensors were secured to the left instep, the right instep, and the lumbar spine using Velcro straps. The EEG system was secured in place using a fitted Neoprene cap aligned with prefabricated holes corresponding to 10–20 EEG system. The electrodes were prepared with conductive electrode gel (SuperVisc 100 gr. HighViscosity Electrolyte-Gel for active electrodes, Brain Products). Data acquisition was communicated wirelessly through the EEG system connected to a laptop computer and recorded through Neuroscan software designed by Neuroelectrics, Inc.

The dual-task standing assessment was comprised of two 60-s trials of standing in each of two conditions: eyes open (i.e., single-task) and eyes open while performing a serial subtraction task (i.e., dual-task) [20]. The serial subtraction task involved audibly counting backward by 3’s from a random 3-digit number between 200 and 999 provided immediately before the trial. During each trial, participants were instructed to keep arms at their side and feet shoulder-width apart. Foot placement was traced in the first trial and this tracing was used in all subsequent trials. Before each trial, participants were reminded to avoid extraneous movements and focus their vision on a small “X” drawn on a wall at eye-level approximately three meters away.

### Postural sway analysis

The instrumented SWAY test was used to assess postural sway with the APDM Mobility Lab software (APDM, Portland, OR). A laptop wirelessly collected data from the sensors at a sampling frequency of 128 Hz and was processed by algorithms developed by the manufacturer to quantify postural sway parameters. Measures of postural sway included mean total sway area (m^2^/s^4^), sway velocity (m/s), and sway path (m/s^2^) were derived from each trial. The dual-task cost to each outcome was computed by calculating the absolute change in each outcome from the single to the dual-task condition [21, 22].

### EEG processing

EEG data was collected with a sampling rate of 500 Hz (Hz) and data was preprocessed and analyzed using the software, CARTOOL [23]. Raw EEG data files were converted by MATLAB into readable format for the CARTOOL software. The data were filtered with DC/Baseline Removal, a Butterworth High Pass Filter at 1 Hz, a Butterworth Low Pass Filter at 80 Hz, a Notch filter at 60 Hz, and then exported into a binary file format to be processed in MATLAB for independent component analysis (ICA) to remove eye blinks and movement artifacts [24]. Electrode impedances were kept below 5 kΩ in all recordings and electrode sites. All electrodes were referred to linked ear lobes, and a ground electrode was attached to the center of the forehead. Noisy channels were identified by visual inspection (standard deviation qualitatively higher than the other measured channels) and interpolated using the nearest-neighbor spline method. Trials with more than eight artifact channels were rejected (8% of trials were rejected from quiet standing and 14% of trials were rejected from dual-tasking). On average, standing conditions had 3.8 ± 0.6 channels rejected whereas dual-tasking conditions had 5.6 ± 2.2 channels rejected. Data were epoched in consecutive two-second windows and any window with noisy data was rejected from the final analysis. Finally, all remaining windows were concatenated into a continuous time series that were used for frequency analysis. Subjects with fewer than 8 s of data were not included in the final frequency analysis [25].

EEG frequency analysis was completed using the CARTOOL spectral analysis function. Spectral analysis is the change of signal power, recorded in microvolts, in the frequency domain. Frequency records the number of oscillations per second in specific bands of interest [26]. We calculated the mean absolute power density (μV^2^/Hz) of alpha (8–16 Hz), theta (4–7 Hz), and beta (18–32 Hz) by using fast Fourier transformation (FFT) by ‘Neuromapping-3,55’ (MBN, Russia). EEG frequencies alpha, theta, beta, power, and theta/beta power ratio were examined with region-of-interest (ROI) analyses. In accordance with Bohle et al. [27], we demarcated six ROIs, anterior left (AL) (F7, Fp1, F3, FC1, FC5, and AF3), central left (CL) (C3, CP1, CP5, and T7), posterior left (PL) (P7, P3, PO1, and PO3), anterior right (AR) (F4, Fp2, F8, FC2, FC6, and AF4), central right (CR) (C4, T8, CP2, and CP6), and posterior right (PR) (P8, P4, O2, and PO4).

### Statistical analysis

Statistical analyses were performed using JMP Pro 14 software (SAS Institute, Cary, NC). Descriptive statistics (i.e., mean and standard deviation (SD)) were used to summarize the demographic characteristics of participants and study outcomes. Shapiro–Wilk tests and histograms were used to examine if the EEG outcomes and postural sway metrics were normally distributed. Variables that were not normally distributed were log transformed prior to modeling. For the EEG outcomes, we excluded outliers defined by data points more than four standard deviations away from the mean of that variable.

First, as participants completed the same protocol during four different visits, we examined the test–retest reliability of EEG-derived metrics during single and dual-task conditions using intraclass correlation coefficient (ICC) analysis [28]. ICC was interpreted as follows: greater than 0.70 was excellent, 0.60 to 0.69 was good, 0.40 to 0.59 was fair, and less than 0.40 was poor [29].

Second, the effect of dual-tasking on EEG power across frequency bands was examined using mixed model repeated measures analysis. Data points from all four visits were included in the analysis. Mixed effects models included condition (single vs dual tasking) was included as a fixed effect and a random intercept for subject. Separate analyses were completed for each dependent variable; that is, absolute alpha, theta, and beta power, and theta/beta ratio.

The relationship between each EEG outcome from each region of interest and each postural sway metric during standing and dual-tasking were assessed by using Pearson’s *r* correlations for the means of the 4 visits within each participant. The results were interpreted as follows: greater than 0.70 was strong, 0.50 to 0.70 was moderate, and 0.30 to 0.50 was weak [30]. The significance level was set to *p* < 0.05 for all analyses.

## Results

Participant characteristics are presented in Table 1.

**Table 1:** Demographic characteristics

Variables	Older adults (n = 30)
Age (years)	73.1 ± 5
Sex (female)	13
MoCA	25.8 ± 3
Height (meters)	1.6 ± 0.1
Weight (kg)	77 ± 18
TUG (s)	12 ± 3
Sway velocity during standing (m/s)	0.32 ± 0.2
Sway velocity during dual-tasking (m/s)	0.36 ± 0.26
Sway area during standing (m^2^/s^4^)	0.05 ± 0.05
Sway area during dual-tasking (m^2^/s^4^)	0.11 ± 0.17
Sway path during standing (m/s^2^)	6.45 ± 2.5
Sway path during dual-tasking (m/s^2^)	9.99 ± 3.86

The results are presented as mean ± standard deviation except for sex variable

MoCA: Montreal Cognitive Assessment, TUG: Timed Up and Go test

### Test–retest reliability of EEG outcomes

Table 2 summarizes the test–retest reliability analysis of the EEG outcomes. The power in nearly all frequency bands and regions-of-interest derived from quiet standing trials demonstrated excellent test–retest reliability over the four separate visits. The test–retest reliability of these metrics derived from dual-task trials was generally moderate in strength.

**Table 2:** Intraclass correlation coefficient results for power bands

Region of interest	Power spectrum and condition							
	Alpha Power, Standing ICC (95% CI)	Alpha Power, Dual-tasking ICC (95% CI)	Theta/beta Power Ratio, Standing ICC (95% CI)	Theta/beta Power Ratio, Dual-tasking ICC (95% CI)	Theta Power, Standing ICC (95% CI)	Theta Power, Dual-tasking ICC (95% CI)	Beta Power, Standing ICC (95% CI)	Beta Power, Dual-tasking ICC (95% CI)
AL	0.68 (0.51–0.81)	0.55 (0.37–0.73)	**0.77** (0.63–0.87)	**0.72** (0.57–0.84)	0.64 (0.47–0.78)	0.57 (0.39–0.74)	**0.73** (0.59–0.84)	0.58 (0.39–0.74)
AR	**0.77** (0.64–0.87)	0.65 (0.47–0.79)	**0.74** (0.59–0.85)	0.65 (0.47–0.79)	**0.77** (0.63–0.86)	0.56 (0.37–0.73)	**0.70** (0.55–0.82)	0.69 (0.51–0.82)
CL	**0.71** (0.56–0.83)	0.64 (0.47–0.78)	**0.77** (0.63–0.87)	0.64 (0.46–0.78)	**0.71** (0.55–0.83)	0.69 (0.52–0.81)	**0.70** (0.54–0.81)	0.46 (0.27–0.67)
CR	**0.76** (0.62–0.86)	0.67 (0.50–0.81)	**0.73** (0.58–0.84)	**0.73** (0.58–0.85)	**0.76** (0.63–0.86)	0.68 (0.51–0.81)	**0.78** (0.65–0.87)	0.66 (0.48–0.80)
PL	**0.77** (0.63–0.86)	0.61 (0.43–0.77)	**0.75** (0.61–0.85)	0.60 (0.41–0.77)	**0.73** (0.58–0.84)	0.66 (0.49–0.80)	0.69 (0.53–0.81)	0.65 (0.47–0.80)
PR	**0.77** (0.63–0.86)	0.61 (0.42–0.77)	**0.76** (0.63–0.86)	**0.71** (0.56–0.83)	0.68 (0.52–0.81)	0.56 (0.37–0.73)	**0.73** (0.59–0.84)	**0.72** (0.56–0.84)

AL: Anterior left, AR: Anterior right, CL: Central left, CR: Central right, PL: Posterior left, PR: Posterior right. Bold numbers indicate ICC > 0.70

### Effect of dual-tasking on brain activity

**Fig. 1:**
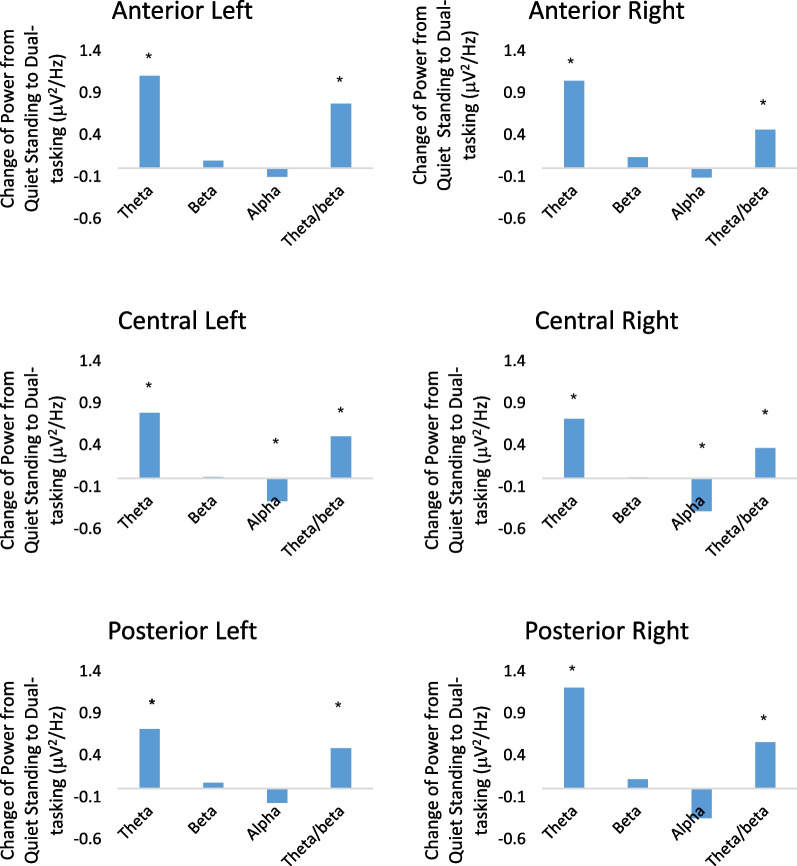
The change (absolute change from quiet standing to dual-task standing condition) of theta, beta, alpha, and theta/beta power ratio bands from standing to dual-tasking condition in the anterior right, anterior left, central right, and posterior right regions. Theta and theta/beta power ratio bands demonstrated an increase from standing to dual-tasking, whereas the alpha band decreased. *p < 0.05

**Fig. 2:**
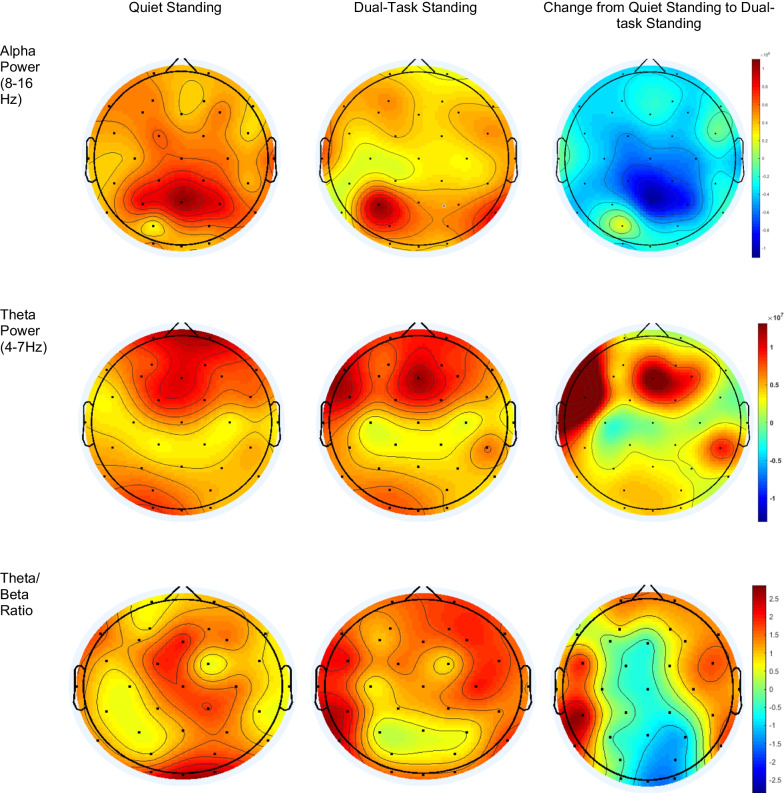
Topographical distribution of selected EEG power spectra during standing and dual-tasking. Warmer or cooler colors indicate more or less power in each frequency band. The topographical distribution of the absolute spectral power (μV^2^/Hz) of alpha demonstrated a significant decrease in the central regions and theta and theta/beta ratio demonstrated a significant increase in all regions

Dual-tasking induced specific changes in brain activity. Table 3 provides the mean and standard deviation of all assessed EEG metrics during standing and dual-tasking in each region of interest which were averaged over the four visits. The results of this analysis demonstrated that compared to quiet standing, dual-tasking induced a significant reduction of alpha power in the central left (p = 0.007), central right (p < 0.001), and posterior left (p = 0.003) regions. Participants exhibited increased theta power in all regions and theta/beta power ratio in the anterior left (p < 0.001), anterior right (p = 0.001), central right (p = 0.003), and posterior right (p = 0.007) regions. No change (p > 0.05) was observed in beta power. Figures 1 and 2 demonstrate the change of alpha power, theta power, and beta power from standing to dual-tasking in each region of interest.

**Table 3:** Values of power bands across the conditions

Region of interest	Power spectrum and condition											
	Alpha power			Theta/beta ratio			Theta power			Beta power		
	standing	Dual-tasking	p-value	standing	Dual-tasking	p-value	standing	Dual-tasking	p-value	standing	Dual-tasking	p-value
Anterior-left	2.3 $\pm$ 2.2	2.3 $\pm$ 2.8	0.71	3.1 $\pm$ 2.2	3.8 $\pm$ 2.5	< 0.001	2.1 $\pm$ 1.9	3.7 $\pm$ 1.0	< 0.001	0.8 $\pm$ 0.5	0.9 $\pm$ 1.3	0.17
Anterior-right	2.1 $\pm$ 1.9	2.1 $\pm$ 2.8	0.58	3.1 $\pm$ 2.3	3.6 $\pm$ 2.5	< 0.001	1.9 $\pm$ 1.7	3.4 $\pm$ 1.0	< 0.001	0.7 $\pm$ 0.5	0.9 $\pm$ 1.5	0.08
Central-left	2.2 $\pm$ 2.2	2.0 $\pm$ 2.4	0.04	2.7 $\pm$ 2.5	3.3 $\pm$ 2.9	< 0.001	1.5 $\pm$ 1.3	2.7 $\pm$ 0.8	0.009	0.7 $\pm$ 0.5	0.7 $\pm$ 1.0	0.88
Central-right	2.3 $\pm$ 2.4	1.9 $\pm$ 2.9	0.003	2.7 $\pm$ 2.3	3.2 $\pm$ 2.4	< 0.001	1.6 $\pm$ 1.5	2.6 $\pm$ 0.9	0.01	0.7 $\pm$ 0.5	0.7 $\pm$ 1.3	0.61
Posterior-left	2.6 $\pm$ 2.4	2.6 $\pm$ 2.6	0.64	3.1 $\pm$ 2.3	3.6 $\pm$ 3.1	0.012	1.8 $\pm$ 1.3	2.9 $\pm$ 0.5	< 0.001	0.7 $\pm$ 0.5	0.9 $\pm$ 0.8	0.05
Posterior-right	2.9 $\pm$ 2.8	2.6 $\pm$ 3.2	0.06	3.1 $\pm$ 2.3	3.6 $\pm$ 3.1	0.004	1.9 $\pm$ 1.6	3.7 $\pm$ 1.3	< 0.001	0.8 $\pm$ 0.6	1.0 $\pm$ 1.5	0.11

Mean and standard deviation of power bands (μV^2^/Hz). P-values were derived from the mixed model analysis

### Correlation analysis between EEG outcomes and postural sway

Participants who exhibited a greater increase in theta/beta power ratio in the anterior left (r = 0.56, p < 0.01) and central right (r = 0.52, p < 0.01) regions from quiet standing to dual-task exhibited a greater dual-task cost (i.e., absolute increase, indicative of worse performance) to postural sway path length. In addition, participants who exhibited greater increase in the theta/beta power ratio in the anterior left (r = 0.52, p < 0.01), central right (r = 0.53, p < 0.01), and posterior left (r = 0.45, p < 0.01) regions showed greater dual-task cost of postural sway area. Lastly, participants who exhibited greater alpha power in the anterior-right (r = 0.52, p < 0.01) and central-right (r = 0.48, p < 0.01) regions had greater postural sway velocity during dual-tasking (Table 4). No other significant relationships were observed between EEG outcomes and postural sway metrics.

**Table 4:** Relationships between EEG outcomes and postural sway

Measurement	Region	Condition	Sway path	Sway area	Sway velocity
Theta/beta ratio	AL	Dual-task cost	0.56*	0.52*	0.28
	AR		0.34	0.57*	0.14
	CL		0.39	0.46*	0.12
	CR		0.52*	0.53*	0.18
	PL		0.29	0.45*	0.05
	PR		0.48*	0.44*	0.15
Alpha	AL	Dual-tasking	0.01	0.11	0.37*
	AR		0.12	0.23	0.52*
	CL		− 0.11	0.11	0.42*
	CR		− 0.06	0.16	0.48*
	PL		− 0.08	0.15	0.39*
	PR		− 0.01	0.20	0.44*

Correlations between EEG theta/beta and alpha power and postural sway during quiet standing, as well as beta and theta power and postural sway metrics during quiet standing and dual-tasking, did not reach statistical significance

AL: Anterior left, AR: Anterior right, CL: Central left, CR: Central right, PL: Posterior left, PR: Posterior right; *p < 0.01

## Discussion

This study demonstrated that EEG alpha, theta, beta powers, and theta/beta power ratio can be reliably measured during standing with and without the concurrent performance of a serial subtraction dual-task in older adults without overt illness or disease. In this cohort, dual-tasking induced a decrease in alpha power from quiet standing to dual-tasking in the central regions and an increase in theta/beta power ratio all regions. Moreover, the degree of change in theta/beta ratio within anterior regions significantly and specifically correlated with the magnitude of dual-task cost to postural control.

The current results indicate that EEG alpha power and theta/beta power ratio are moderate-to-highly reliable metrics that are sensitive to changes in brain activity induced by performing a verbalized serial subtraction task while standing in older adults. A previous study reported that alpha and theta powers demonstrated strong test–retest reliability during cognitive testing and resting-state in young adults [31]. In our cohort of older adults with an average age of 73, EEG outcomes derived from a standing balance assessment also demonstrated strong test–retest reliability. However, ICC levels were slightly lower during dual task conditions. It is possible that during dual-tasking, the level of task difficulty and cognitive demand differed from individual to individual, and/or within an individual between visits, which might have led to increased variability in brain activity as measured by EEG. The verbalization of serial subtraction during dual-tasking might have also been a source of noise in the EEG recording that also caused reduced reliability across repeated assessments. Our analytic methods, including independent component analysis and careful visualization of the data, nevertheless led to at least moderate test–retest reliability in this condition. That said, future studies are needed to investigate whether titration of a cognitive task and non-verbal responses might lead to better ICC results during dual-tasking in older adults.

In line with our hypothesis, we observed dual-tasking induced reductions in alpha band power in central areas across both hemispheres. Previous studies reported decreased power in the alpha frequency band with increasing balance task difficulty in electrode-based regions of interest [32, 33]. For instance, Hülsdünker et al. reported that decreases in alpha power were strongest in the sensorimotor areas with increasing balance task demands [33]. The reduction of alpha power has been associated with decreased motor inhibition during increased postural task difficulty. A systematic review investigating corticospinal activity during dual-tasking reported decreased motor inhibition when an additional cognitive task is added to quiet standing [34]. It has been shown that an additional task may result in a cognitive-first strategy in older adults and lead to worse motor performance by reducing the ability to activate motor inhibitory networks [14]. In the current study, we did not investigate the effects of dual tasking on serial subtraction performance, nor the relationship between serial subtraction performance and brain activity, because it is difficult to fully characterize serial subtraction performance within and between participants. Moreover, most participants committed only a small number of response errors during the task and there was thus relatively little variance in related performance metrics between participants. Future work is warranted that uses other cognitive tasks (e.g., the Auditory Stroop or N-back) tested in both single and dual-task conditions, to examine the influence of prioritization on dual-task performance and related brain activity.

Dual-tasking, compared to quiet standing, also resulted in an increased theta/beta power ratio in older adults. Several recent studies have investigated cortical dynamics of standing balance and reported modulated brain activity at different frequency bands during challenging balance conditions. Studies in young adults demonstrated increased theta band power and decreased beta band power over the frontal and sensorimotor cortical regions, suggesting that this could be due to increased attentional control required to monitor postural stability and maintain balance during dual-tasking [35, 36]. Theta rhythm has also been demonstrated to increase while individuals are engaged in challenging balance conditions including perturbations, visual occlusion, and additional cognitive load [35, 37, 38]. In addition, beta activity suppression was observed during gait control and challenging balance tasks [36, 37, 39]. It is possible that increased theta and decreased beta power are both involved during active postural control and may fluctuate as task difficulty changes. One way to better understand these fluctuations is to investigate theta/beta ratio during challenging balance tasks. Theta/beta ratio has shown to be a robust measure of attentional control and strongly correlates with EEG event-related potential (ERP) P3 latency [40]. The P3, a positive peak that appears with a latency between 250 to 500 ms in the ERP, has been implicated in attention and working memory processes [41]. Investigating the change of theta/beta ratio might provide a better understanding of the change of attentional control by the effect of dual-tasking in older adults.

When a cognitive task is performed during standing, the two tasks compete for shared and limited cognitive resources [42]. In older adults, as compared to young adults, standing appears to require more cognitive resources, potentially to compensate for age-related impairments in the postural control system [10, 43]. Competition of shared cognitive resources during dual-task standing thus leads to cognitive-motor interference and dual-task “costs” to postural control and/or cognitive task performance [44]. Moreover, in older adults, the increased dual-task cost is predictive of future falls and cognitive decline [45]. The present study demonstrated that the change of theta/beta ratio from single-task standing to dual-task standing significantly correlated with postural sway area and sway path in older adults. It is possible that the capacity of the brain to dual-task is reflected by the extent of change in the theta/beta power ratio. Our results suggest that the underlying neurophysiological mechanism might be linked to increased attentional control from standing to dual-tasking which can be indicative of worse balance outcomes in older adults.

EEG has low spatial resolution compared to other neuroimaging and neurophysiological tools [46]. Although we did an ROI analysis, the location of the channels might not correspond to the activity of the underlying brain structure. Future studies are needed to implement source localization with high-density EEG and subject-specific MRI to aid localizing accuracy. Nevertheless, this study suggests that in older adults, dual-tasking while standing decreases alpha power which is associated with motor inhibition and increases theta/beta power ratio which links to attentional control compared to quiet standing, and that a greater increase in an EEG marker of attentional control is associated with worse dual-task standing performance. Future studies are needed to understand whether modulating EEG alpha power or theta/beta power ratio may improve dual-task standing performance and reduce risk of falls in older adults.

## Data Availability

Data and materials can be made available upon reasonable request to the authors.
